# Movement disorders in neuroleptic-naïve patients with schizophrenia spectrum disorders

**DOI:** 10.1186/s12888-014-0280-1

**Published:** 2014-10-09

**Authors:** Moges Ayehu, Teshome Shibre, Barkot Milkias, Abebaw Fekadu

**Affiliations:** Department of Psychiatry, Hawassa University, College of Medicine and Health Sciences, Hawassa, Ethiopia; Department of Psychiatry, Addis Ababa University, College of Health Sciences, School of Medicine, Addis Ababa, Ethiopia; University of Toronto, Ontario Shores Center for Mental Health Sciences, Toronto, Canada; Department of Psychological Medicine, Centre for Affective Disorders, King’s College London, Institute of Psychiatry, London, UK

**Keywords:** Parkinsonism, Dyskinesia, Spontaneous movement disorders, Dyskinetic movements, Antipsychotics, Psychotic disorder, Schizophrenia, Schizophreniform disorder, Schizoaffective disorder, Ethiopia, Developing country

## Abstract

**Background:**

Spontaneous Movements Disorders (SMDs) or dyskinetic movements are often seen in patients with schizophrenia and other psychotic disorders, and are widely considered to be adverse consequences of the use of antipsychotic medications. Nevertheless, SMDs are also observed in the pre-neuroleptic ear and among patients who were never exposed to antipsychotic medications. The aim of this study was to determine the extent of SMDs among antipsychotic-naïve patients in a low income setting, and to evaluate contextually relevant risk factors.

**Methods:**

The study was a cross-sectional facility-based survey conducted at a specialist psychiatric hospital in Addis Ababa, Ethiopia. Consecutive consenting treatment-naïve patients with a diagnosis of schizophrenia, schizoaffective disorder and schizophreniform disorder contacting services for the first time were assessed using the Simpson-Angus Rating Scale (SAS) and the Abnormal Involuntary Movement Scale (AIMS) to evaluate the presence of SMDS. Scale for the Assessment of Negative Symptoms (SANS) and Scale for the Assessment of Positive Symptoms (SAPS) were administered to evaluate negative and positive symptom profiles respectively. Body mass index (BMI) was used as a proxy measure for nutritional status.

**Result:**

Sixty-four patients, 67.2% male (n = 43), with first contact psychosis who met the DSM-IV-TR criteria for schizophrenia (n = 47), schizophreniform disorder (n = 5), and schizoaffective disorder (n = 12) were assessed over a two month study period. Seven patients (10.9%) had SMDs. BMI (OR = 0.6, 95% CI = 0.40, 0.89; p = 0.011) and increasing age (OR = 1.10; 95% CI = 1.02, 1.20; p = 0.017) were associated with SMD.

**Conclusions:**

This finding supports previous suggestions that abnormal involuntary movements in schizophrenia and other psychotic disorders may be related to the pathophysiology of psychotic disorders and therefore cannot be attributed entirely to the adverse effects of neuroleptic medication.

## Background

Different neurological motor abnormalities, especially dyskinesia and parkinsonism, have been reported in patients with schizophrenia [1-5]. With the introduction of antipsychotic medications and their numerous neurological adverse effects, these neurological abnormalities were generally presumed to be consequences of treatment with these newly introduced medications [6]. However, a wide range of neurological motor disturbances has been reported in patients with schizophrenia who have never been treated with antipsychotic drugs, pre-dating the antipsychotic-era [7-10]. For example, research in the past two decades has identified spontaneous dyskinesia in a proportion of drug-naïve patients with schizophrenia [11-14]. Spontaneous movement disorders (SMD), including tardive dyskinesia-like syndrome, were reported in 4% to 11% of patients with no prior treatment history with antipsychotic medications [10-17]. In a recent review of 13 studies, the median rate of spontaneous dyskinesia was 9%, with spontaneous parkinsonism being the commoner abnormality [5]. Muscle rigidity and bradykinesia, often referred to together as akinetic type signs, are reported to be the most common parkinsonian signs in drug-naïve patients and occur more often than non-akinetic type signs such as tremor, glabellar tap, and salivation [12,18-22]. Non-akinetic parkinsonism becomes more severe following treatment with antipsychotics than the akinetic type and it has been suggested that it may be primarily a drug-induced phenomenon [21,22]. The most commonly reported dyskinetic symptoms are involuntary orofacial movements [8,14]. SMDs have also been reported in relatives of those with schizophrenia and dyskinesia, as well as in subjects with schizophrenia spectrum personality conditions, especially in schyzotypal personality [17,23,24].

The main factors reported to have significant association with SMD were patients’ age, psychopathology, pre-morbid functioning, educational achievement, and IQ [12,14,19-22,25-31]. In addition, there is preliminary evidence that pre-existing abnormal movements increase the risk of subsequent extrapyramidal side effects.

Based on the above observations, it has been proposed that idiopathic extrapyramidal disturbances may be intrinsic to the pathophysiology of schizophrenia and that antipsychotic medications may act by modifying the expression of disease-based motor dysfunctions [32-34], perhaps through specific alterations in subcortical dopamine neuronal activity due to basal ganglia dysregulation and dysfunction in cortical-basal, ganglia-cortical circuitry [18]. It is unclear how much ‘neuroleptic-induced’ motor disturbance should actually be attributed to the natural history of psychosis, aging, or other nondrug causes. Identifying this may be relevant to reducing negative attitude towards the side effects of antipsychotic medications and enhancing our understanding of the pathophysiology of psychosis. At present, nearly all patients with psychotic disorders in high income countries are exposed to antipsychotic medications [25]. In low income countries like Ethiopia, up to 90% of patients may not be exposed to antipsychotic medications [35,36], providing an opportunity to explore the prevalence and determinants of SMD among patients not exposed to antipsychotic treatments.

There are no published data from Ethiopia reporting on the prevalence of extrapyramidal symptoms or side effects; however, the incidence and prevalence of these symptoms are likely to be comparable to what has been reported in the broader literature. There are at least three outstanding issues that need addressing, which may be potentially relevant to our knowledge of extrapyramidal symptoms: 1) The contribution of antipsychotics above and beyond the natural risk of movement disorders is unclear; 2) the prevalence of movement disorders in treatment-naïve patients is not well documented in low income settings; 3) it has not yet been established whether there are unique risk factors more relevant for low income settings, such as malnutrition, infection and trauma.

### Objectives

The primary objective of the study was to assess the extent of SMDs among antipsychotic-naïve patients with schizophrenia spectrum disorders (schizophrenia, schizoaffective and schizophreniform disorder) attending the outpatient department of a psychiatric hospital in Ethiopia. We additionally aimed to determine the association of socio-demographic and clinical factors with SMDs, including the occurrence of potential risk factors that may be contextually relevant, such as nutritional status.

## Methods

### Study design and setting

The study was a facility-based cross-sectional survey conducted at a psychiatric hospital located in the capital city, Addis Ababa. The hospital is the oldest and largest public psychiatric hospital in Ethiopia. Patients nationwide come to the hospital to receive treatment for severe mental disorders. The hospital service is organized under specialist programs or specialist case teams and each case team is expected to have expert knowledge about the disorders it treats. Patients with psychotic disorders are treated by psychoses case teams. These case teams manage all individuals with psychosis referred or seen by the hospital.

### Participants

Participants of this study were patients aged between 18 and 60 years with a clinical diagnosis of schizophrenia, schizoaffective and schizophreniform disorders with no previous antipsychotic treatment or current treatment with medications that could induce extrapyramidal symptoms such as metoclopramide or other antiemetics. Other drugs that may induce extrapyramidal symptoms such as amoxapine and buspirone were not considered because they were not available in the Ethiopia market. Persons above the age of 60 were excluded because of the concern that older age may increase the risk of movement disorders as well as the potential for somatic and neurological diseases. Additionally, patients with catatonia were excluded as catatonia may be confused with other movement abnormalities.

### Assessment

Successive patients were screened from the triage unit by a final-year psychiatry resident and consenting patients fulfilling inclusion criteria were offered a detailed assessment. Assessments consisted of an evaluation of basic socio-demographic characteristics, diagnosis, symptom severity, physical health status and occurrence of SMDs. Nutritional status, history of serious head injury and birth trauma were evaluated as contextually relevant risk factors. All assessments were made by the same final-year psychiatry resident. A diagnosis of schizophrenia, schizoaffective disorder or schizophreniform disorder was confirmed using a diagnostic check list from the Text Revision of the Diagnostic and Statistical Manual of Mental Disorders [37]. The presence of extrapyramidal symptoms was evaluated using Simpson-Angus Scale (SAS) [38] and Abnormal Involuntary Movement Scale (AIMS) [39]. The SAS is a 10-item scale and has been shown to have good sensitivity [38]. The extrapyramidal symptoms assessed by the SAS are rigidity, hypersalivation, glabellar reflex, and tremor. On most items, severity is scored from 0 (none) to 4 (severe). A score of 1 usually indicates the presence of the extrapyramidal symptoms in a mild form. The commonly used threshold value to diagnose parkinsonism is 0.3 or more, which is used as a cut-off score to confirm occurrence of parkinsonism in the current report. The AIMS is commonly used to evaluate dyskinetic symptoms. The 12 questions of the AIMS cover anatomical locations of the dyskinetic movements (face, extremities, and trunk), global severity of the dyskinesia, global judgment of incapacitation and awareness of abnormality. The AIMS is administered through a clinician interview and physical examination. Scores on AIMS range from 0 (absent) to 5 (severe). Conventionally, dyskinesia is present, according to the AIMS scale, when there are mild movements in at least two areas or moderate movements in at least one area [40].

Patient symptoms profile and severity were assessed using the Scale for the Assessment of Positive Symptoms (SAPS) and Scale for the Assessment of Negative Symptoms (SANS) [41,42]. Both scales were designed to provide a detailed assessment of positive and negative symptoms of schizophrenia and can be used separately or in tandem. The domains assessed in the SAPS include hallucinations, delusions, bizarre behavior, and thought disorders, while the domains assessed in the SANS include affective flattening, poverty of speech, apathy, anhedonia, and inattentiveness. Nutritional status of participants was measured by the Qutelet’s body mass index (BMI), a measure often used to define nutritional status in adults. It is estimated as weight in kilograms divided by height in metres squared. The BMI is easy to measure and closely relates to individuals’ food consumption levels. The BMIs of the participants were categorized according to the recommendations of the WHO Expert Committee for chronic energy deficiency [43]. According to this WHO classification, participants with BMI values of less than 16 were considered to have severe energy deficiency; those with scores of 16–16.9 to have moderate deficiency; and those with scores of 17–18.4 to have mild deficiency. Those with BMI values of 18.5-24.9 were considered to be normal. Mid-arm circumference was also assessed as an additional measure of nutritional status. Finally, use of substances of potential abuse (alcohol, khat, cigarettes and cannabis) was assessed. The assessment considered initially whether the participant had lifetime or current use of these substances and those reporting use in the past year were questioned in more detail about the frequency and quantity of the substances used.

### Data management

Data were collected by a final-year psychiatry resident who received a half-day of training in addition to the extensive residency training he had received. Data were entered into the Statistical Package for Social Sciences, Version 15 for Windows for analysis. The focus of the analyses was on simple descriptive outputs (frequencies, percentages, means and standard deviations). Normality of continuous variables was checked visually using histogram and tested using Kolmogorov-smirnov test. For normally distributed data, equality of variance was checked using Leven’s test before applying T-test. The few analyses for comparison and associations used the Independent Sample T-test and logistic regression analysis. Because of the small number of cases, only bivariate associations were explored.

### Ethical considerations

The proposal was approved by the Scientific Committee of the Department of Psychiatry, School of Medicine, College of Health Sciences, Addis Ababa University and the Ethics Committee of Amanuel Specialised Mental Hospital. Interviews were conducted in private after obtaining informed consent. Analysis was conducted on anonymised data. Any new clinically relevant information generated in the research interview was passed on to the clinical team for the benefit of the participant.

## Result

### Socio-demographic and clinical characteristics of the participants

Sixty-four patients, mostly men (n = 43/64; 67.2%), with first contact psychosis were assessed over a 3 month period (April 01- June 30, 2013) prior to exposure to antipsychotic or other psychotropic medications. The age of participants was between 18 and 60 years and the mean (SD) age was 29.2 (9.5). Most participants were single (44/64; 68.8%) and had a diagnosis of schizophrenia (n = 47; 73.4%). The duration of illness prior to presentation (duration of untreated psychosis) was between 1 month and 156 months, with a median (IQR) of 24 months (12, 57 months). Details are provided in Table 1.

**Table 1:** **Distribution of patients by socio-demographic characteristics**

**Characteristics**		**Number**	**Percent**
Sex	Male	43	67.2
	Female	21	32.8
Age (in years)	18-24	28	43.8
	25-34	13	20.3
	35 and above	23	35.9
Educational status	Not able to read and write	10	15.6
	Basic writing and reading upto grade 6	21	32.8
	Above grade 6	33	51.5
Religion	Orthodox Christian	37	57.8
	Muslims	22	34.4
	Others	5	7.8
Marital status	Married	11	27.3
	Single	44	68.8
	Divorced or separated	9	14.1
Ethnicity	Amhara	20	31.3
	Oromo	16	25.0
	Guragae	16	25.0
	Others	12	18.8
Occupation	Unemployed	27	42.2
	Self-employed	14	21.9
	Farmer	14	21.9
	Student/house wife	9	14.1
Living arrangement	Living with parent or sibling	52	81.3
	Living alone or with own family	12	18.7
Diagnosis	Schizophrenia	47	73.4
	Schizophreniform disorder	5	7.8
	Schizoaffective disorder	12	18.8
Duration of illness	≤1 year	23	35.9
	1-2 years	11	17.2
	2-4 years	14	21.8
	4-13 years	16	25.0

### Spontaneous movement disorders or dyskinetic movements

Based on standard cut-off points used to make a diagnosis of movement disorders, seven cases (10.9%) were found to have spontaneous movement disorders. Four participants had parkinsonism while one had dyskinesia and two had both parkinsonism and dyskinesia. Four of those with SMD were female and three male. Although a proportionately higher number of women had SMD, this difference was not statistically significant (Fisher’s exact p = 0.204). Those with SMD were older, with a mean (SD) age of 38.3 (12.5) years compared with participants without SMD who had a mean age of 28.1 (8.6) years. This difference was statistically significant (mean difference = 10.2; t = 2.8; p = 0.006). These differences are reflected in the mean scores for both the SAS and AIMS scales, which were relatively higher among older participants, women and those with more chronic illness (Table 2). The topography of dyskinetic movements was primarily orofacial, while the parkinsonian symptoms were found in the elbow, shoulder and arm regions, followed by wrist and leg regions.

**Table 2:** **The mean, minimum and maximum Simpson-Angus Rating Scale and the Abnormal Involuntary Movement Scale scores of participants by demographic and clinical characteristics**

**Characteristics**		**Simpson-Angus (SAS) score**			**Abnormal Involuntary Movements (AIMS) score**		
		**Mean**	**Minimum**	**Maximum**	**Mean**	**Minimum**	**Maximum**
Gender	Male	0.4	0.0	8.0	0.3	0.0	12.0
	Female	1.43	0.0	11.0	0.8	0.0	10.0
Age	<24	0.4	0.0	11.0	0.2	0.0	6.0
	25-34	0.3	0.0	4.0	0.9	0.0	12.0
	≥ 35	1.4	0.0	11.0	0.4	0.0	10.0
BMI	≥ 18.5	0.2	0.0	4.0	0.0	0.0	0.0
	<18.5	1.4	0.0	11.0	1.0	0.0	12.0
Duration of illness (in months)	1-12	0.4	0.0	6.0	0.0	0.0	0.0
	13-24	0.2	0.0	2.0	0.0	0.0	0.0
	25-48	0.3	0.0	4.0	0.0	0.0	0.0
	49-156	2.0	0.0	11.0	1.8	0.0	12.0
Diagnosis	Schizophrenia	0.9	0.0	11.0	0.6	0.0	12.0
	Schizophreniform disorder	1.2	0.0	6.0	0.0	0.0	0.0
	Schizoaffective disorder-bipolar type	0.0	0.0	0.0	0.0	0.0	0.0
	Schizoaffective disorder- depressive type	0.0	0.0	0.0	0.0	0.0	0.0

### Nutritional status and substance use behavior

**Figure 1:**
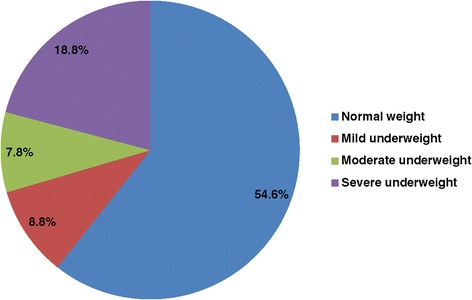
**Proportion of participants with evidence of malnutrition according to the BMI-based WHO classification.**

Based on the WHO Expert Committee classification for Chronic Energy Deficiency in adults, close to half of the participants (n = 29; 45.3%) were in the underweight range with a BMI value of less than 18.5. Among those with low BMI values, 12 (41.4%) had BMI values of less than 16, which is classified as severely underweight. These details are presented in Figure 1. Thirty six (56.3%) participants had either current or past history of substance use (alcohol, khat, cigarettes or cannabis) and among those 52.4% of those used substances daily.

### Exploratory analysis of associated factors

Because of the relatively small sample size and the small number of cases with SMD, results presented are based on a bivariate model. Increasing age was associated with SMD (OR = 1.10; 95% CI = 1.02, 1.20; p = 0.017). An inverse association was found between patients’ nutritional status and the presence of spontaneous movement disorders; therefore increasing body weight (a proxy measure of dietary health), assessed with BMI, was inversely associated with SMD (OR = 0.6; 95% CI = 0.4, 0.8; p =0.01). The mean score on the Simpson-Angus scale was also marginally associated with the SANS score (β = .25; 95% CI = 0.0, 0.06; p = 0.049).

## Discussion

This is the first study on this subject in Ethiopia and is also one of few research reports on this subject from Africa. We evaluated a total of sixty-four patients with first contact psychosis who met the DSM-IV-TR criteria for schizophrenia, schizophreniform, and schizoaffective psychosis over the period of the study. The principal finding of this study is the confirmation that movement disorders occur in persons with schizophrenia spectrum disorders prior to exposure to antipsychotic medications, which are often presumed to be the cause of movement disorders in those with psychotic disorders. In this study at least one in ten treatment-naïve individuals (10.9%; 7/64) had spontaneous movement disorders. Most of those with SMD (6/7) had parkinsonism, while nearly half (3/7) had dyskinesia either alone or with parkinsonian movement disorders. The prevalence of spontaneous movement disorders in our study (10.9%) is consistent with the prevalence finding of SMDs in previous studies [15], particularly that of spontaneous parkinsonism [12,17,18], which lies between 4% and 21%. Rigidity and motor slowness were the common manifestations of parkinsonism in our study, consistent with previous studies [12,18-22] which reported akinetic symptoms, such as rigidity and bradykinesia, much more frequently than non-akinetic type movement disorders, such as tremor, glabelar tap, and salivation in antipsychotic-naïve patient with parkinsonian movement disorders. Consistent with previous studies [8,14], the commonly found dyskinestic symptoms in our study were orofacial movements. Thus, the findings in our study support the assertion that spontaneous movement disorders may be partly a disease-induced state-dependent change, independent of the effect of antipsychotic medications.

One of the incidental findings of this study is the high rate of protein energy malnutrition: 45.5% (n = 29) of participants had BMI value under 18.5, which was significantly higher among those with spontaneous movement disorders, where 6/7 of those with SMD had low BMI. Alternatively, this could suggest that higher BMI was protective of SMD. The small number of cases prohibits in-depth speculation about the relevance of this finding. However, one postulation may be whether this finding may have relevance to the aetiology of schizophrenia.

The aetiology of schizophrenia and other psychotic disorders remains largely undefined despite extensive research on possible biological mechanisms. Genetic constitution is important, but environmental factors like an unhealthy lifestyle with a poor diet may be involved. One area of focus regarding environmental risk factors has been around the role of polyunsaturated fatty acids. Low maternal fish and seafood consumption during pregnancy has been reported to increase the risk for a low IQ and suboptimal neuro-developmental outcomes in childhood [44], factors that in turn are associated with an increased risk for adult mental disorders like schizophrenia [45]. Research done by Horrobin [46] and Fenton [47] reported lower levels of polyunsaturated fatty acids (PUFA) in brain content, red blood cells and skin fibroblasts among patients with schizophrenia, compared with a healthy population. Hedelin et al. [48] also found that a high dietary intake of fish, omega-3 and omega-6 PUFA, as well as vitamin D, may have a protective effect against positive psychotic-like symptoms. However, we did not find studies reporting association between poor nutrition and SMD among persons with schizophrenia. This association may be attributed to specific alterations in subcortical dopamine neuronal activity [18]. Further studies are required to determine whether poor nutrition is a risk factor for SMDs.

The other factor significantly associated with SMD was increasing age. Although some previous studies did not find such an association [4], increasing age is often considered an important risk factor for SMD [9,49]. Additionally, there was weak association between negative symptoms and parkinsonian movement disorder. Exploratory analysis did not find an association between duration of illness, gender and SMD, which is consistent with previous reports [4].

### Limitation of the study

The limitations of cross-sectional studies hold true also for this study. The number of recruited patients was relatively small and only patients who came to the hospital were recruited, limiting generalisability. Because of the small sample size, the analysis for association between potential risk factors and SMD should be considered exploratory.

## Conclusions

This study supports the argument that involuntary movements (and parkinsonian symptoms) in schizophrenia may be, at least in part, intrinsic to the pathophysiology of the illness rather than a side effect of its treatment. Nutrition may simply be a contextual risk factor or represent a factor linked to the pathophysiology of the illness. Further studies need to confirm these observations and systematically examine the relationship between SMDs and these risk factors with a larger sample size and more robust methodology. Additionally, determining the course of SMDs and their impact on treatment would also be of interest.
